# Patterns of Ocular Involvement and Associated Factors in Adult Measles: A Retrospective Study from a Romanian Tertiary Hospital

**DOI:** 10.3390/clinpract16010004

**Published:** 2025-12-25

**Authors:** Dragoș Ștefan Lazăr, Adina-Alexandra Nanu, Ilie-Andrei Condurache, Maria Nica, Catrinel Tudosie, Maria Alexandra Malciolu-Nica, Alexandra Ioana Grigore, George Sebastian Gherlan, Corneliu Petru Popescu, Simin Aysel Florescu

**Affiliations:** 1 Department of Infectious Diseases, “Carol Davila” University of Medicine and Pharmacy, 020021 Bucharest, Romania; dragos.lazar@umfcd.ro (D.Ș.L.); maria.nica@umfcd.ro (M.N.); catrinel.gurgui@rez.umfcd.ro (C.T.); george.gherlan@umfcd.ro (G.S.G.); corneliu.popescu@umfcd.ro (C.P.P.); simin.florescu@umfcd.ro (S.A.F.); 2 “Dr. Victor Babes” Clinical Hospital of Infectious and Tropical Diseases, 030303 Bucharest, Romania; ilie.condurache@spitalulbabes.ro (I.-A.C.); alexandra.malciolu@spitalulbabes.ro (M.A.M.-N.); ioana.grigore@spitalulbabes.ro (A.I.G.)

**Keywords:** inflammation, keratitis, measles, ocular complications, neutrophil-to-lymphocyte ratio (NLR)

## Abstract

Background: Measles re-emergence has been reported across Europe, with Romania being among the most affected countries in 2023–2024. Although ocular manifestations are recognized in measles, their frequency and inflammatory correlates in hospitalized adults have not been well characterized. Methods: This study retrospectively analyzed the medical records of adults treated for laboratory-confirmed measles at a Bucharest hospital between July 2023 and July 2024. Data from specialist eye examinations were used to compare patients with keratitis against those with other ocular issues. Results: A total of 250 adult patients were included. Of the 88 patients referred for ophthalmologic examination, 93.2% showed ocular lesions. Keratitis was the primary form, identified in 64.6% of these cases. Patients with keratitis had blood markers indicating a more activated inflammatory profile (higher neutrophile-to-lymphocytes ratio). Pneumonia and respiratory failure were not associated with ocular lesion status; inflammatory markers were more strongly linked to respiratory failure than to ocular involvement. Conclusions: Ocular lesions were highly prevalent in hospitalized adult measles cases during the 2023–2024 Romanian epidemic wave, and keratitis was common. Ocular involvement correlated with mucosal disease expression and systemic inflammatory activation. Systematic ophthalmologic assessment should be considered during measles epidemic peaks to improve early identification of clinically relevant ocular complications.

## 1. Introduction

Measles is an acute, highly contagious viral disease caused by a virus of the genus *Morbillivirus*, family *Paramyxoviridae* [1]. It is transmitted via airborne droplets, and its basic reproduction number (R_0_) is often cited to be 12–18, meaning that in a fully susceptible population one single case may infect 12–18 other individuals [2,3]. Clinically, measles is characterized by fever, maculopapular rash, and respiratory symptoms, but can frequently be complicated by pneumonia, otitis media, encephalitis, or even death [4].

Koplik’s spots, considered pathognomonic for measles, generally appear 1–2 days before the onset of the cutaneous rash and may persist for 1–2 days after its emergence [5,6]. Another important feature is keratoconjunctivitis, which can become severe and, if untreated, may contribute to vision loss or blindness, particularly in malnourished or immunocompromised patients [4].

Histopathologically, Koplik’s spots reflect small foci of epithelial necrosis in the buccal mucosa, accompanied by an inflammatory infiltrate. Within these lesions, one can identify multinucleated giant cells, known as Warthin–Finkeldey cells, which are considered a hallmark of measles infection [7,8]. Similar giant cells are also found in the respiratory tract and in lymphoid tissues such as lymph nodes, spleen, and tonsils, reflecting the same viral cytopathic effect [8]. Thus, the tiny bluish-white spots seen on the oral mucosa mirror represent, at the microscopic level, the broader histopathological signature of the disease.

Histopathological changes are also evident in the conjunctiva during measles infection. The conjunctivitis, often striking and accompanied by photophobia and lacrimation, corresponds microscopically to epithelial necrosis and a dense inflammatory infiltrate. Multinucleated giant cells, similar to those found in Koplik’s spots and in the respiratory epithelium, may also be observed in the conjunctival tissue [7,8]. In severe cases, especially in malnourished children, corneal involvement with ulceration and keratomalacia can occur, which contributes to the risk of blindness [4,9]. Thus, both Koplik’s spots and measles-related conjunctivitis share a common histopathological substrate, reflecting the virus’s predilection for mucosal epithelia.

The disease has been known since antiquity, with Rhazes in the 9th century providing the first clear description distinguishing measles from smallpox [7]. Before vaccination became available, measles was one of the leading causes of childhood morbidity and mortality worldwide. In the pre-vaccine era, the disease caused between 2 and 2.6 million deaths annually, mostly among children [4,8]. In 1980 alone, before the widespread rollout of immunization programs, an estimated 2.6 million measles deaths occurred globally [4].

The first live attenuated measles vaccine was developed in the early 1960s, with Enders and Katz reporting successful immunization in 1960 and 1962 [9]. Its widespread introduction in 1963 dramatically reduced both cases and deaths worldwide [10,11]. However, to achieve herd immunity, vaccination coverage must remain at or above 95% with two doses of measles-containing vaccine [12].

In Romania, a major measles epidemic started in 2016, linked to a decline in vaccination coverage. By mid-2019, more than 17,500 measles cases and 64 deaths had been reported since 2016 in Romania [13]. Subsequently, totals reached more than 20,000 cases and 64 deaths by August 2020 [14]. After a temporary decline, cases surged again, and in December 2023 the Ministry of Health officially declared a national measles epidemic [15,16]. By 2024, Romania had become the most affected country in the European region, with more than 30,000 measles cases reported [16].

During the same period, several European countries—including the United Kingdom, Austria and Germany—also reported substantial increases in measles transmission, with outbreaks linked predominantly to under-vaccinated communities. Romania remained one of the most affected countries in Europe, with 29,968 cases reported from July 2023 to July 2024, of which 21,762 were laboratory-confirmed, 7268 epidemiologically linked and 938 clinically diagnosed [17].

Routine measles vaccination in Romania is included in the National Immunization Program and relies on the two-dose MMR schedule (administered at 12 months and 5 years of age). However, vaccination coverage has declined over the last decade, falling below the 95% herd-immunity threshold, contributing to sustained viral circulation and recurrent epidemic waves [18].

In this epidemiological context, adult measles cases represent an increasingly visible component of the disease burden, reflecting historical gaps in vaccination coverage and ongoing viral circulation. Despite this shift toward adult involvement, data on organ-specific complications—particularly ocular manifestations—in hospitalized adults remain limited.

The present work set out to evaluate the appearance of eye lesions in adult patients with measles in relation to other clinical manifestations encountered during the course of the disease.

## 2. Materials and Methods

### 2.1. Study Design and Setting

This was a retrospective observational study conducted at “Dr. Victor Babeș” Clinical Hospital for Infectious and Tropical Diseases, Bucharest, Romania (VBH). The study period covered all measles cases admitted between July 2023 and July 2024.

### 2.2. Study Population

This retrospective cohort included all consecutive adult patients admitted with laboratory-confirmed measles at VBH over a one-year study period. The study was performed in a tertiary care hospital that treats both adult and pediatric patients with infectious diseases. Only patients aged ≥ 18 years were eligible for inclusion in the analysis; pediatric patients were not included in this study. Patients were hospitalized based on clinical suspicion and epidemiological context, and measles confirmation was established using serum IgM detection according to national guidelines.

This retrospective analysis included only hospitalized adult patients with laboratory-confirmed measles. Patients who were diagnosed with measles but managed in outpatient settings or did not require admission were not captured in the hospital database and therefore could not be included in the study.

Ophthalmologic consultation was performed by a specialist ophthalmologist for patients who underwent eye examination during hospitalization. Ophthalmologic examination was performed selectively based on clinical judgement of the attending infectious disease specialist. This non-systematic referral approach may have resulted in missed mild/asymptomatic lesions and limits any inference on the true prevalence of ocular involvement in the full cohort. We have acknowledged this as a limitation.

Ophthalmologic consultations were requested at the discretion of the treating physician and could be performed at any time during hospitalization; precise timing in relation to hospital admission or symptom onset was not consistently documented.

### 2.3. Variables and Definitions

Clinical, epidemiological, laboratory, and imaging data were extracted from electronic medical records. Pneumonia was defined as clinically suspected lower respiratory tract infection with radiological confirmation documented in the medical chart. Respiratory failure was defined as the presence of hypoxemia requiring supplemental oxygen therapy or ventilatory support, as documented by the treating physicians. Keratitis and other ocular lesions were confirmed by ophthalmologic examination performed during admission. “Other ocular lesions” included conjunctivitis, non-specific ocular surface inflammation, and other non-corneal findings documented by the ophthalmologist during examination. The ophthalmic examination was performed using slit-lamp biomicroscopy (Topcon SL-D2 Slit Lamp, Tokyo, Japan), employing a focused light beam and a high-magnification microscope to examine the eye’s anterior structures. It uses adjustable illumination, optics, and filters (including cobalt blue and fluorescein) to assess morphology, transparency, and lesions, with optional fluorescein staining for epithelial detail. Koplik’s sign was recorded as present or absent based on physical examination on admission.

Data regarding measles vaccination history, nutritional status (including vitamin A levels), smoking status, alcohol consumption, and detailed comorbidity profiles were not consistently available in the medical records and were therefore not included in the analysis.

### 2.4. Data Sources

Data were extracted from the observation charts and electronic medical records of all eligible patients hospitalized in the Infectious and Tropical Diseases wards of the “Dr. Victor Babeș” Hospital. Laboratory parameters included in the analysis reflect the values obtained at the time of hospital admission.

### 2.5. Statistical Analysis

All data were analyzed using IBM SPSS Statistics for Windows, Version 25.0. USA -Armonk, NY: IBM Corp. Categorical variables were expressed as counts and percentages, and group differences were assessed using Fisher’s exact test. Continuous variables were presented as means with standard deviations or medians with interquartile ranges, depending on distribution. Normality was assessed using the Shapiro–Wilk test. For continuous variables with non-parametric distribution, group comparisons were performed using the Mann–Whitney U test. The significance level was set at α = 0.05.

Given the exploratory nature of the study and the limited size of some subgroups, multivariable modeling was not performed.

### 2.6. Ethics Approval

This study was conducted in accordance with the principles of the Declaration of Helsinki. Ethical approval for this retrospective analysis was obtained from the Ethics Committee of “Dr. Victor Babeș” Clinical Hospital for Infectious and Tropical Diseases, Bucharest, Romania (approval No.: 10605, date of approval: 16 June 2025).

## 3. Results

As shown in Table 1, a total of 250 patients with measles were included in the analysis. The mean age was 33.3 ± 10.7 years (median 33, IQR 23–43), and 52.4% were female. Most patients were living in urban areas (60.0%) and had health insurance coverage (72.8%). Among 239 patients with available employment data, 10.9% worked in the healthcare sector. Regarding educational level (*n* = 198), 53.0% possessed secondary education, while 27.3% had no formal schooling or only a primary education. Recent travel history (past 14 days) was rare (0.8%). Antibiotic exposure in the preceding 14 days was reported in 29.6%. Only one patient (0.4%) had a documented previous history of measles.

The median time from symptom onset to hospital admission was 5 days (IQR 4–6). Fever was present in 96.8% of cases, and all patients presented with maculopapular exanthema; Koplik’s spots were documented in 64.0%. The median interval between rash onset and admission was 2 days (IQR 1–3).

Complications were frequent. Pneumonia occurred in 86.4% of patients, respiratory failure in 16.4%, and encephalitis in 0.4%. Hepatic involvement, indicated by elevated serum transaminases (ALT, AST > 50 UI/mL), occurred in the majority of patients (79.6%) and presented as mild to moderate hepatitis. The mean length of hospitalization was 4.6 ± 1.7 days (median 4, IQR 4–6). Antibiotic therapy during hospitalization was administered in 65.6% of patients.

Laboratory parameters demonstrated mean neutrophil and lymphocyte counts of 4649.6 ± 1942.6 cells/μL and 640 ± 466.1 cells/μL, respectively, resulting in a mean neutrophil-to-lymphocyte ratio (NLR) of 9.86 ± 7.59 (median 8.0). Mean AST and ALT levels were 164.3 ± 167.7 UI/mL and 225.7 ± 232.4 UI/mL, respectively; the mean ALT/AST ratio was 1.44 ± 0.84.

Of the 250 hospitalized adults with measles, 88 patients (35.2%) were referred for ophthalmologic evaluation based on clinical suspicion during hospitalization; the remaining patients did not undergo formal ophthalmologic examination. Ophthalmology referral occurred at the discretion of the treating team and timing varied during admission. Ocular lesions were documented in 93.2% of those examined, with keratitis reported in 64.6%.

Among the 88 patients who underwent ophthalmological evaluation, ocular lesions were present in 82 cases (93.2%). Comparative analyses between patients with and without ocular lesions did not identify statistically significant differences across demographic, clinical, laboratory, or outcome parameters (all *p* > 0.05). The proportion of female patients was similar in both groups (50.0% vs. 46.3%). Median age did not differ substantially (35 vs. 34 years). Urban residence was recorded in 50.0% of patients without ocular lesions and 62.2% of those with lesions.

No differences were observed regarding symptom onset-to-admission intervals, the presence of fever, Koplik sign, or exanthema–admission intervals. Complication rates were comparable, including pneumonia (83.3% vs. 91.5%) and respiratory failure (0% vs. 15.9%), and no cases of encephalitis were reported in either subgroup. Median length of hospitalization was similar between groups (4 vs. 5 days).

Median neutrophil counts, lymphocyte counts, NLR, AST, ALT, and ALT/AST ratio did not significantly differ between groups. The study did not reveal a link between ocular lesions and hepatitis. ALT and AST elevations showed no significant association with ocular involvement (*p* = 0.556 and *p* = 0.245, respectively). Antibiotic treatment rates were comparable (66.7% vs. 67.1%). The preceding findings are presented in Table 2. Considering the very small number of patients without ocular lesions (N = 6), results presented in Table 2 must be visualized as a descriptive summary and *p*-values are presented only for numerical context.

Comparisons between patients with and without ocular lesions should be interpreted with caution, as the group without ocular findings was very small (*n* = 6), resulting in limited statistical power.

Table 3 compares patients’ characteristics according to the type of ocular lesions. Of 82 patients with ocular lesions, 53 (64.6%) had keratitis; the remaining cases were mainly conjunctivitis, blepharitis or non-specifical lesions. Keratitis patients differed from those with other ocular lesions on several statistically significant measures, whereas sex distribution and age were comparable. Among the 78 patients with available employment data, there was no difference in occupational categories between groups, and healthcare workers predominated in both (85.7% vs. 88.0%). Urban residence was reported in 66.0% of keratitis cases versus 55.2% of other ocular lesions.

Regarding educational level (*n* = 65), differences were observed in distribution: absence of formal education were more common among those with keratitis. Symptom onset-to-admission intervals were similar across groups.

Clinical findings were differentially distributed: fever was documented in 89.7% vs. 100% of patients, and Koplik’s sign was significantly more frequent in the keratitis group (44.8% vs. 84.9%). Pneumonia was identified in all patients with other ocular lesions compared to 86.8% of those with keratitis. Rates of respiratory failure did not differ significantly. Median hospitalization duration was 5 days in both groups. Antibiotic treatment rates during admission were similar (72.4% vs. 64.2%).

Laboratory analyses showed lower median lymphocyte counts (600 vs. 400 cells/μL) and higher median NLR values (7.66 vs. 10.0) among patients with keratitis. Median AST, ALT, and ALT/AST ratios were comparable between groups.

The distribution of educational level differed between patients with keratitis compared to those with other ocular lesions. Primary education and absence of formal education were more frequent in the keratitis group (15.4% and 17.9%, respectively), whereas academic education was more common among patients with other ocular lesions (42.3% vs. 20.5%). Secondary education represented the largest category in both groups, but was more frequent in patients with non-keratitis lesions (53.8% vs. 46.2%).

**Figure 1:**
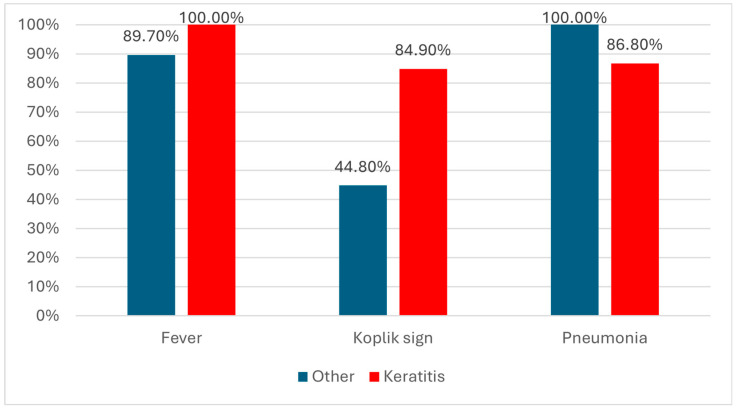
Distribution of the patients with ocular lesions according to the existence of fever, Koplik sign, pneumonia and type of lesions.

As illustrated in Figure 1, the distribution of selected clinical features differed between patients with keratitis and those with other ocular lesions. Fever was reported in all patients with keratitis (100%) compared to 89.7% in the non-keratitis group. Koplik’s spots were also more frequently documented in the keratitis subgroup (84.9% vs. 44.8%). Conversely, pneumonia was present in all patients with other ocular lesions (100%) compared with 86.8% of those with keratitis.

**Figure 2:**
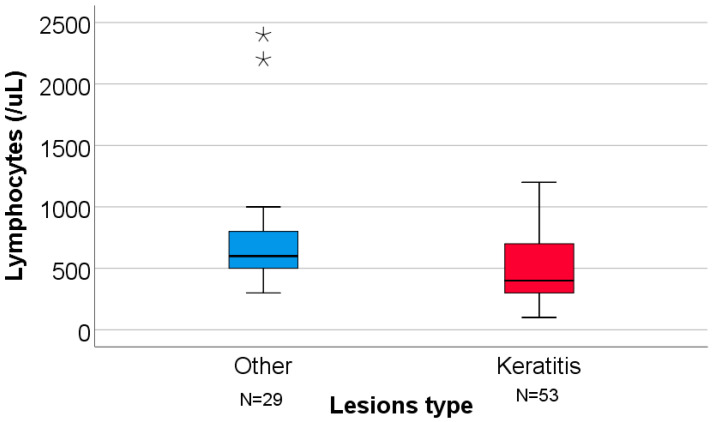
Lymphocyte values by ocular lesion type (keratitis vs. other lesions). For the illustration of the quantitative values distributions in the box-plot graphs, the IBM SPSS Statistics software illustrates any values that are above the 3rd quartile (75th percentile) + 3*interquartile range, as extreme outliers represented by asterisk symbols in the graph.

Figure 2 shows the distribution of lymphocyte counts according to the type of ocular lesions. Median values were comparable between groups, with significantly lower lymphocyte counts observed in patients with keratitis.

**Figure 3:**
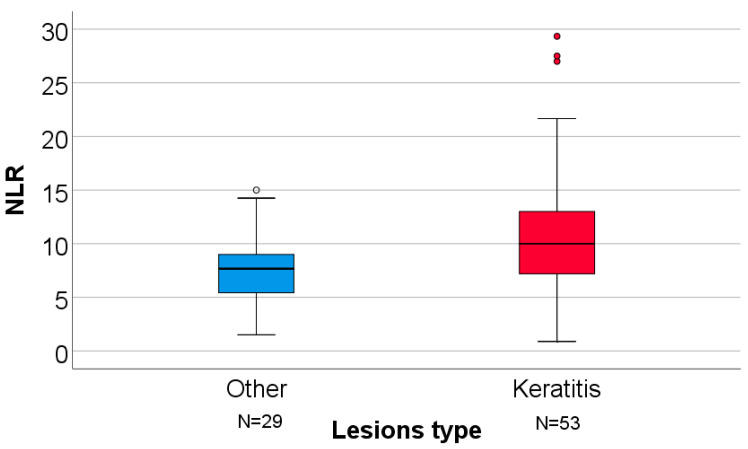
Comparison of NLR values by ocular lesion type (keratitis vs. other lesions). NLR = Neutrophil-to-lymphocyte ratio. For the illustration of the quantitative values distributions in the box-plot graphs, the IBM SPSS Statistics software illustrates any values that are above the 3rd quartile (75th percentile) + 1.5*interquartile range, as outliers represented by circles in the graph.

Figure 3 illustrates the distribution of neutrophil-to-lymphocyte ratio (NLR) in patients with keratitis and other ocular lesions. NLR values showed greater dispersion among patients with keratitis, with several high-value outliers, while observing significantly higher values in comparison to patients with other lesions. Further data concerning the analysis of ocular lesion type can be found in the Supplementary data.

**Figure 4:**
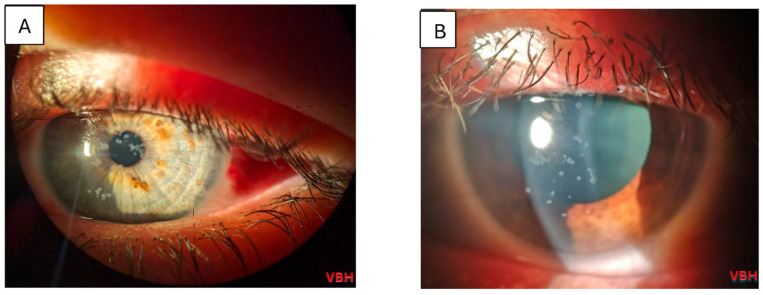
Slit lamp examination of the left eye in a 23 years old patient with measles reveals classic superficial punctate keratitis with coalescent lesions towards the center of the cornea and subconjunctival hemorrhage (**A**); slit lamp examination of the left eye in a 33 years old patient reveals classic superficial punctate keratitis (**B**). Photo: Malciolu–Nica M.A (VBH).

**Figure 5:**
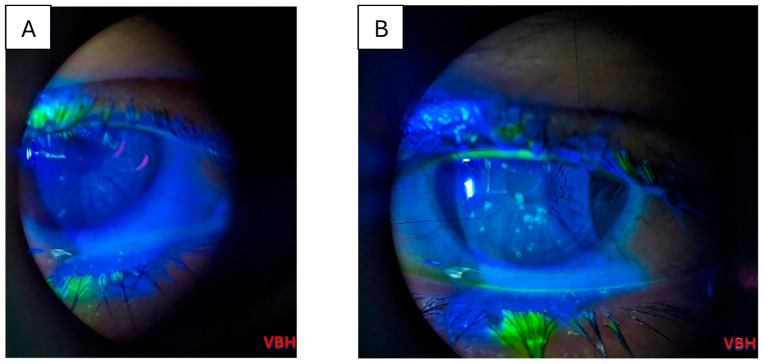
Slit lamp examination in a 20 years old patient with measles, under blue cobalt light with fluorescein staining, revealing mild superficial punctate keratitis spread all over the corneal epithelium (**A**); slit lamp examination in a 28 years old patient with measles, under blue cobalt light with fluorescein staining, revealing superficial punctate keratitis coalescent lesions towards the center of the cornea (**B**). Green coloration results from the combination of cobalt blue illumination and orange fluorescein uptake by devitalized epithelium. Photo: Malciolu–Nica M.A (VBH).

**Figure 6:**
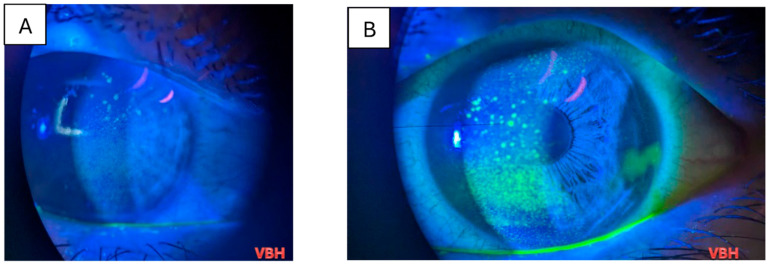
Slit lamp examination of the right eye in a 21 years old patient with measles, under blue cobalt light with fluorescein staining, revealing corneal superficial punctate eruptions and inferior discrete corneal erosion (**A**); slit lamp examination of the left eye revealing superficial punctate keratitis and severe corneal erosions inferiorly (**B**). Green coloration results from the combination of cobalt blue illumination and orange fluorescein uptake by devitalized epithelium. Photo: Malciolu–Nica M.A (VBH).

**Figure 7:**
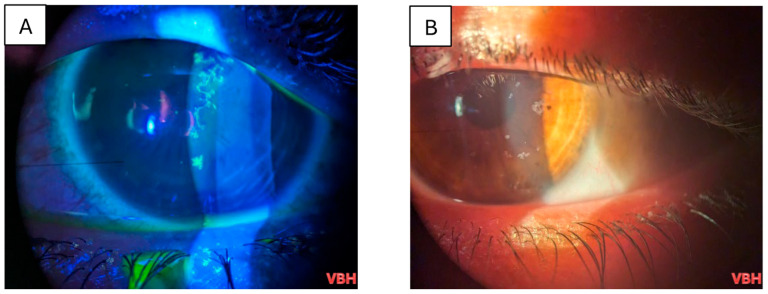
Slit lamp examination of the right eye in a 28 years old patient with measles, under blue cobalt light with fluorescein staining, revealing superficial punctate eruptions and 2 dendritic herpetic lesions vertically spread on the corneal epithelium (**A**); Slit lamp examination of the left eye revealing superficial punctate keratitis (**B**). Green coloration results from the combination of cobalt blue illumination and orange fluorescein uptake by devitalized epithelium. Photo: Malciolu–Nica M.A (VBH).

**Figure 8:**
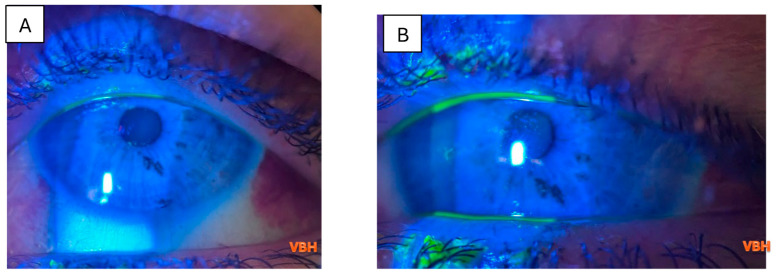
Slit lamp examination of the right eye in a 23 years old patient with measles, under blue cobalt light with fluorescein staining, revealing corneal superficial punctate eruptions and 2 small dendritic herpetic lesions centrally, and nasal subconjunctival hemorrhage (**A**); slit lamp examination of the left eye revealing superficial punctate keratitis and 3 small dendritic lesion and temporal subconjunctival hemorrhage (**B**). Green coloration results from the combination of cobalt blue illumination and orange fluorescein uptake by devitalized epithelium. Photo: Malciolu–Nica M.A.

Below we illustrate aspects observed in the ophthalmologic examinations conducted. We observe conjunctival hyperemia with follicles and hemorrhages; corneal epitheliopathy is suggested by mild fluorescein staining under cobalt blue illumination, with no stromal or anterior chamber involvement (Figure 4, Figure 5, Figure 6, Figure 7 and Figure 8).

**Figure 9:**
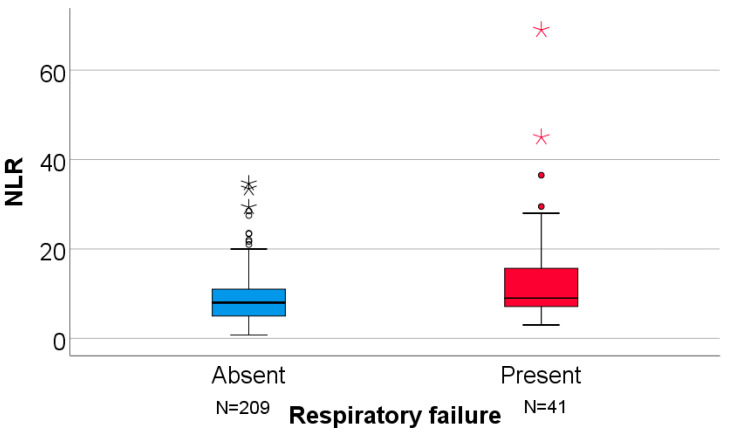
Comparison of the patients’ NLR value according to the existence of respiratory failure. NLR = Neutrophil-to-lymphocyte ratio. For the illustration of the quantitative values distributions in the box-plot graphs, the IBM SPSS Statistics software illustrates any values that are above the 3rd quartile (75th percentile) + 1.5*interquartile range, as outliers represented by circles in the graph. As for values that are above the 3rd quartile (75th percentile) + 3*interquartile range, the software represents the values as extreme outliers represented by asterisk symbols in the graph.

In certain situations, in our study we observed dendritic lesions suggestive of herpes reactivation, which can occur in the context of measles. Consequently, mixed ocular findings may appear, as depicted in Figure 8 and Figure 9.

Table 4 summarizes the comparison of patient characteristics according to the presence of Koplik’s sign. Sex distribution, age, workplace domain, living environment, educational level, travel history, prior antibiotic exposure, health insurance status, and history of previous measles infection did not differ significantly between patients with positive and negative Koplik’s sign (all *p* > 0.05).

The median time from symptom onset to admission was slightly shorter among patients with Koplik’s sign (median 5 days vs. 5 days, IQR 4–5 vs. 4–7; *p* = 0.027). Fever was also more frequently documented in the Koplik-positive subgroup (98.8% vs. 93.3%; *p* = 0.027). No significant differences were observed regarding exanthema-to-admission intervals. Rates of pneumonia, respiratory failure, and encephalitis were comparable between groups. Median hospitalization duration did not differ significantly. Median neutrophil counts, lymphocyte counts, NLR, AST, ALT, and ALT/AST ratio showed no significant differences between patients with and without Koplik’s sign.

Table 5 shows the comparison of patient characteristics according to the presence of pneumonia. Demographic variables—including sex, age, workplace domain, living environment, and educational level—did not differ significantly between patients with and without pneumonia. No significant differences were observed regarding recent travel, prior antibiotic exposure, health insurance status, or prior measles history.

Symptom timing parameters (onset-to-admission and exanthema-to-admission intervals) were similar in both groups, as was fever frequency. As expected, respiratory failure was more frequently observed among patients with pneumonia (18.5% vs. 2.9%; *p* = 0.023).

Encephalitis was rare in both groups. Median hospitalization duration tended to be slightly longer in patients with pneumonia, although this difference did not reach statistical significance.

Median neutrophil counts, lymphocyte counts, NLR, AST, ALT, and ALT/AST ratio did not differ significantly by pneumonia status. Antibiotic treatment during admission was more common among patients with pneumonia (68.5% vs. 47.1%; *p* = 0.019).

In Table 6 we summarize the comparison of patients with and without respiratory failure. Female sex was less frequent among patients with respiratory failure (29.3% vs. 56.9%; *p* = 0.002). Patients with respiratory failure were slightly older (median 37 vs. 32 years; *p* = 0.049). Workplace domain, environment, educational level, travel history, prior antibiotic exposure, health insurance coverage, and prior measles history did not differ significantly between groups.

The interval from symptom onset to admission was longer among patients with respiratory failure (median 5 days, IQR 4–7 vs. 5 days, IQR 3–6; *p* = 0.034). Fever was present in the majority of both groups. Median hospitalization duration did not differ significantly.

Laboratory parameters showed significantly higher median neutrophil counts in patients with respiratory failure (5200 vs. 4100 cells/μL; *p* < 0.001) and higher NLR values (median 9.0 vs. 8.0; *p* = 0.025). Lymphocyte counts, AST, ALT, and ALT/AST ratio were comparable.

Antibiotic treatment was more frequently administered in patients with respiratory failure (85.4% vs. 61.7%; *p* = 0.004). Most commonly used antibiotics included amoxicillin/clavulanate and ceftriaxone.

Figure 9 shows the distribution of NLR values according to respiratory failure status. Patients with respiratory failure demonstrated higher NLR values and more extreme outliers compared with those without respiratory failure.

## 4. Discussion

In this cohort of adults with measles admitted during Romania’s 2023–2024 epidemic wave, we observed no significant sex difference, with a median age of 33 years. As a retrospective study, we were unable to assess IgG antibodies to confirm reinfection; however, World Bank vaccination coverage data show estimates ranging from 78% to 91% between 1988 and 1994 [19]. When overlaid with the current epidemic wave, these coverage gaps may partly explain the relatively high number of cases among young adults. This observation reinforces that past vaccination gaps may reemerge during future measles epidemics.

In our study, we identified a substantial number of patients with ocular involvement and the majority of them had keratitis. This proportion is substantially higher than typically described in the literature from non-epidemic or high-resource settings, where ocular complications, particularly keratitis, are considered uncommon [1]. One likely contributing factor is that ophthalmologic evaluation is not routinely performed in all hospitalized measles patients in most published studies. Our findings support the hypothesis that the ocular burden of measles may be underestimated if not actively screened [20,21].

The strong association between keratitis and the presence of Koplik’s sign observed in this study suggests that keratitis may reflect a more intense mucosal viral cytopathic effect. Koplik’s spots themselves are considered the clinical correlate of epithelial necrosis with multinucleated giant cells—the same histopathological substrate described in measles-related ocular involvement. To our knowledge, this correlation has not previously been quantified in a hospitalized adult epidemic cohort.

Patients with keratitis also showed lower lymphocyte counts and higher NLR values, suggesting that ocular involvement may align with a more activated inflammatory profile. This observation is in line with recent Romanian data describing inflammatory hematologic abnormalities in hospitalized adult measles cases [22]. Whether ocular findings might be used as a clinical marker of systemic inflammatory intensity in measles outbreaks warrants further investigation.

It remains unclear whether ocular involvement can reliably indicate a more severe overall disease course, and this is something that deserves further study. We also found that pneumonia did not substantially change how often keratitis occurred compared to other ocular lesions. Still, in the small subgroup with non-keratitis eye findings, every patient also had pneumonia. This suggests that some ocular changes may be secondary to severe respiratory complications rather than a direct viral effect on the ocular surface.

This study has limitations: it is single-center, retrospective, and ophthalmologic examination was not performed in all patients. Because examinations were requested based on the clinical judgment of the treating physician rather than performed systematically, selection bias is very likely. Patients with more evident ocular symptoms were preferentially examined; therefore, the reported prevalence of ocular lesions likely overestimates the true burden among all hospitalized measles cases and should not be generalized.

Vaccination status was frequently not documented and could not be used for stratification; similarly, no evidence of possible reinfection was available based on systematic measles IgG testing.

The diagnosis of measles was based on clinical and epidemiology-driven data, and serological IgM testing was performed for confirmation. IgM assays may sometimes give false positive results in outbreak situations. However, all the patients included in the analysis presented a compatible clinical picture and epidemiological exposure before laboratory confirmation was obtained. To this end, IgM positivity was interpreted in conjunction with clinical and epidemiological data, and we are confident that the probability of misclassification of cases in our cohort is low.

Systematic microbiological testing for respiratory pathogens was not available for all patients; pneumonia was defined based on clinical and radiological criteria.

No structured ophthalmologic follow-up was available after discharge; therefore, long-term visual outcomes could not be assessed. These factors limit causal inference. Nevertheless, this study highlights the importance of structured ophthalmologic evaluation during measles epidemic peaks.

We did not have access to outpatient measles cases; therefore, findings apply only to hospitalized adults. Because the study population was limited to hospitalized patients, the findings may not be generalizable to milder measles cases managed in outpatient settings.

Detailed data regarding smoking status, alcohol consumption, and other comorbidities were not consistently available in the electronic medical records and could therefore not be included in the analysis. The potential influence of these factors on systemic inflammation or ocular involvement could not be assessed and represents an additional limitation of the study. The association between educational level and keratitis should be interpreted cautiously, given small cell sizes and potential residual confounding.

Because ophthalmologic consultation could be requested at any time during hospitalization, the exact timing of ocular assessment relative to admission or symptom onset was variable and not consistently available, representing an additional limitation. Given the exploratory nature of the analyses and the number of comparisons performed, the findings should be interpreted cautiously and considered hypothesis-generating.

Overall, keratitis was common in hospitalized adult measles patients in VBH during the 2023–2024 epidemic wave. Ocular involvement correlated with mucosal disease expression and inflammatory activation, suggesting that ophthalmologic manifestations may represent a clinically relevant phenotype during periods of intense viral circulation.

Based on our data, patients presenting both Koplik’s sign and a NLR ≥ 9.75 had a 95% positive predictive value for keratitis (overall accuracy 64.6%). These two criteria could support a practical screening approach to prioritize ophthalmologic referral when resources are limited.

## 5. Conclusions

In this cohort of hospitalized adults with measles during the 2023–2024 epidemic wave in Romania, ocular involvement was frequent and clinically relevant. Keratitis was the dominant phenotype and was associated with the presence of Koplik’s sign and with an inflammatory profile characterized by lower lymphocyte counts and higher neutrophil-to-lymphocyte ratios. These findings suggest that ophthalmologic manifestations may reflect mucosal disease intensity and systemic inflammatory activation, representing a distinct clinical dimension of measles expression. Systematic ophthalmologic assessment during epidemic peaks may improve early recognition of severe ocular involvement and could contribute to more targeted supportive management in hospitalized patients.

## Figures and Tables

**Table 1:** Characteristics of the patients analyzed in the study.

Parameter	Value
N, %	250 (100%)
Sex (Female) (Nr., %)	131 (52.4%)
Age (Mean ± SD, Median (IQR))	33.32 ± 10.65, 33 (23–43)
Workplace domain (Nr., %) (N = 239)	
Healthcare	26 (10.9%)
Other	213 (89.1%)
Environment (Urban) (Nr., %)	150 (60%)
Education (Nr., %) (N = 198)	
Primary	22 (11.1%)
Secondary	105 (53%)
Academic	39 (19.7%)
No education	32 (16.2%)
Travel in the past 14 days (Nr., %) (N = 249)	2 (0.8%)
Antibiotic treatment in the past 14 days (Nr., %)	74 (29.6%)
Health insurance (Nr., %)	182 (72.8%)
Medical history—Measles (Nr., %)	1 (0.4%)
Symptoms	
Days—Onset—Admission (Mean ± SD, Median (IQR)) (N = 249)	4.72 ± 1.69, 5 (4–6)
Fever (Nr., %)	242 (96.8%)
Exanthema (Nr., %)	250 (100%)
Koplik sign (Nr., %)	160 (64%)
Days—Exanthema—Admission (Mean ± SD, Median (IQR)) (N = 243)	1.76 ± 1.61, 2 (1–3)
Complications (Nr., %)	
Pneumonia	216 (86.4%)
Respiratory failure	41 (16.4%)
Encephalitis	1 (0.4%)
Hospitalization period (Mean ± SD, Median (IQR))	4.57 ± 1.72, 4 (4–6)
Laboratory parameters (Mean ± SD, Median (IQR))	
Neutrophils (cells/μL)	4649.6 ± 1942.55 4400 (3300–5625)
Lymphocytes (cells/μL)	640 ± 466.12 500 (400–800)
NLR	9.857 ± 7.588 8 (5.5–11.4)
AST (UI/mL) (N = 241)	164.26 ± 167.7 107 (59–222)
ALT (UI/mL) (N = 249)	225.69 ± 232.42 144 (64–300.5)
ALT/AST (N = 241)	1.44 ± 0.837 1.313 (0.941–1.678)
Antibiotic treatment (Nr., %)	164 (65.6%)
Eye exam (Nr., %)	
Ophthalmologic examination performed	88 (35.2%)
Ocular lesions (N = 88)	82 (93.2%)
Type of lesions (N = 82)	
Keratitis	53 (64.6%)
Other	29 (35.4%)

N = Number of cases, SD = Standard deviation, IQR = Interquartile range, NLR = Neutrophil-to-lymphocyte ratio, ALT = Alanine transaminase, AST = Aspartate transaminase.

**Table 2:** Comparison of patients’ characteristics according to the existence of ocular lesions.

Parameter/Ocular Lesions (N = 88)	Absent (N = 6)	Present (N = 82)	*p*
Sex (Female) (Nr., %)	3 (50%)	38 (46.3%)	1.000 ‡
Age (Median (IQR))	35 (22–41.25)	34 (24–44)	0.759 §
Environment (Urban) (Nr., %)	3 (50%)	51 (62.2%)	0.673 ‡
Education (Nr., %) (N = 69)			
Primary (N = 7)	0 (0%)	7 (10.8%)	0.525 ‡
Secondary (N = 36)	4 (100%)	32 (49.2%)	
Academic (N = 19)	0 (0%)	19 (29.2%)	
No education (N = 7)	0 (0%)	7 (10.8%)	
History—Travel (Nr., %)	0 (0%)	0 (0%)	-
History—Antibiotic treatment (Nr., %)	2 (33.3%)	23 (28%)	1.000 ‡
Health insurance (Nr., %)	4 (66.7%)	65 (79.3%)	0.606 ‡
Medical history—Measles (Nr., %)	0 (0%)	0 (0%)	-
Symptoms			
Days—Onset—Admission (Median (IQR)) (N = 87)	5 (3.25–7)	5 (4–6)	0.726 §
Fever (Nr., %)	6 (100%)	79 (96.3%)	1.000 ‡
Koplik sign (Nr., %)	4 (66.7%)	58 (70.7%)	1.000 ‡
Days—Exanthema—Admission (Median (IQR)) (N = 86)	2 (0–3.25)	2 (1–2)	0.909 §
Complications (Nr., %)			
Pneumonia	5 (83.3%)	75 (91.5%)	0.446 ‡
Respiratory failure	0 (0%)	13 (15.9%)	0.586 ‡
Encephalitis	0 (0%)	0 (0%)	-
Hospitalization period (Median (IQR))	4 (3–4.5)	5 (4–6)	0.103§
Laboratory parameters (Median (IQR))			
Neutrophils (cells/μL)	4300 (3325–4775)	4300 (3300–5550)	0.772 §
Lymphocytes (cells/μL)	600(275–825)	500(400–700)	0.914 §
NLR	8.28 (5.24–12)	8.33 (6.22–11.91)	0.728 §
AST (UI/mL) (N = 85)	241(38–252)	117(64–229)	0.878 §
ALT(UI/mL)	172(37–353)	180(66–360.5)	0.477 §
ALT/AST (N = 85)	1.37 (1.07–1.45)	1.32 (0.92–1.77)	0.835 §
Antibiotic treatment (Nr., %)	4 (66.7%)	55 (67.1%)	1.000 ‡

‡ Fisher’s Exact Test, § Mann–Whitney U Test, N = Number of cases, IQR = Interquartile range, NLR = Neutrophil-to-lymphocyte ratio, ALT = Alanine transaminase, AST = Aspartate transaminase. Due to the very small number of patients without ocular lesions (*n* = 6), results should be interpreted descriptively.

**Table 3:** Comparison of patients’ characteristics according to the type of ocular lesions.

Parameter/Ocular Lesions Type (N = 82)	Other (N = 29)	Keratitis (N = 53)	*p*
Sex (Female) (Nr., %)	13 (44.8%)	25 (47.2%)	1.000 ‡
Age (Median (IQR))	32 (24–45)	34 (24–43.5)	0.973 §
Workplace domain (Nr., %) (N = 78)			
Other (N = 68)	24 (85.7%)	44 (88%)	0.740 ‡
Healthcare (N = 10)	4 (14.3%)	6 (12%)	
Environment (Urban) (Nr., %)	16 (55.2%)	35 (66%)	0.351 ‡
Education (Nr., %) (N = 65)			
Primary (N = 7)	1 (3.8%)	6 (15.4%)	0.019 ‡
Secondary (N = 32)	14 (53.8%)	18 (46.2%)	
Academic (N = 19)	11 (42.3%)	8 (20.5%)	
No education (N = 7)	0 (0%)	7 (17.9%)	
History—Travel (Nr., %)	0 (0%)	0 (0%)	-
History—Antibiotic treatment (Nr., %)	9 (31%)	14 (26.4%)	0.798 ‡
Health insurance (Nr., %)	23 (79.3%)	42 (79.2%)	1.000 ‡
Medical history—Measles (Nr., %)	0 (0%)	0 (0%)	-
Symptoms			
Days—Onset—Admission (Median (IQR)) (N = 81)	5 (4–6)	5 (4–6)	0.964 §
Fever (Nr., %)	26 (89.7%)	53 (100%)	0.041 ‡
Koplik sign (Nr., %)	13 (44.8%)	45 (84.9%)	<0.001 ‡
Days—Exanthema—Admission (Median (IQR)) (N = 80)	2 (1–2)	2 (1–2)	0.776 §
Complications (Nr., %)			
Pneumonia	29 (100%)	46 (86.8%)	0.048 ‡
Respiratory failure	7 (24.1%)	6 (11.3%)	0.204 ‡
Encephalitis	0 (0%)	0 (0%)	-
Hospitalization period (Median (IQR))	5 (4–6)	5 (4–6)	0.698 §
Laboratory parameters (Median (IQR))			
Neutrophils (cells/μL)	4500 (3600–5200)	4000 (3200–5800)	0.516 §
Lymphocytes (cells/μL)	600 (500–800)	400 (300–700)	0.004 §
NLR	7.66 (4.83–9.33)	10 (7.1–13.1)	0.016 §
AST (UI/mL) (N = 80)	118 (78–173)	116 (51–241.5)	0.963 §
ALT (UI/mL)	191 (75.5–277)	179 (58–490.5)	0.907 §
ALT/AST (N = 80)	1.38 (1.03–2)	1.22 (0.92–1.74)	0.360 §
Antibiotic treatment (Nr., %)	21 (72.4%)	34 (64.2%)	0.474 ‡

‡ Fisher’s Exact Test, § Mann–Whitney U Test, N = Number of cases, SD = Standard deviation, IQR = Interquartile range, NLR = Neutrophil-to-lymphocyte ratio, ALT = Alanine transaminase, AST = Aspartate transaminase.

**Table 4:** Comparison of patients’ characteristics according to the existence of positive Koplik sign.

Parameter/Koplik Sign (N = 250)	Negative (N = 90)	Positive (N = 160)	*p*
Sex (Female) (Nr., %)	53 (58.9%)	78 (48.8%)	0.147 ‡
Age (Median (IQR))	30.5 (22–43)	34 (24–43)	0.136 §
Workplace domain (Nr., %) (N = 239)			
Other (N = 213)	74 (87.1%)	139 (90.3%)	0.516 ‡
Healthcare (N = 26)	11 (12.9%)	15 (9.7%)	
Environment (Urban) (Nr., %) (N = 198)	55 (61.1%)	95 (59.4%)	0.893 ‡
Education (Nr., %)			
Primary (N = 22)	6 (8.1%)	16 (12.9%)	0.475 ‡
Secondary (N = 105)	37 (50%)	68 (54.8%)	
Academic (N = 39)	18 (24.3%)	21 (16.9%)	
No education (N = 32)	13 (17.6%)	19 (15.3%)	
History—Travel (Nr., %) (N = 249)	1 (1.1%)	1 (0.6%)	1.000 ‡
History—Antibiotic treatment (Nr., %)	28 (31.1%)	46 (28.7%)	0.773 ‡
Health insurance (Nr., %)	61 (67.8%)	121 (75.6%)	0.186 ‡
Medical history—Measles (Nr., %)	0 (0%)	1 (0.6%)	1.000 ‡
Symptoms			
Days—Onset—Admission (Median (IQR)) (N = 249)	5 (4–7)	5 (4–5)	0.027 §
Fever (Nr., %)	84 (93.3%)	158 (98.8%)	0.027 ‡
Days—Exanthema—Admission (Median (IQR)) (N = 243)	2 (1–3)	1 (1–2)	0.295 §
Complications (Nr., %)			
Pneumonia	78 (86.7%)	138 (86.3%)	1.000 ‡
Respiratory failure	15 (16.7%)	26 (16.3%)	1.000 ‡
Encephalitis	0 (0%)	1 (0.6%)	1.000 ‡
Hospitalization period (Median (IQR))	4.5 (3–6)	4 (4–6)	0.730 §
Laboratory parameters (Median (IQR))			
Neutrophils (cells/μL)	4300(3250–5425)	4550(3300–5700)	0.591 §
Lymphocytes (cells/μL)	600 (400–800)	500 (400–700)	0.294 §
NLR	7.7 (4.75–11)	8.07 (5.75–12)	0.247 §
AST (UI/mL) (N = 241)	100 (59–173)	112 (61–233)	0.195 §
ALT (UI/mL) (N = 249)	142 (48–272)	144 (67–350)	0.163 §
ALT/AST (N = 241)	1.26 (0.92–1.65)	1.33 (0.97–1.69)	0.604 §
Antibiotic treatment (Nr., %)	60 (66.7%)	104 (65%)	0.890 ‡

‡ Fisher’s Exact Test, § Mann–Whitney U Test, N = Number of cases, IQR = Interquartile range, NLR = Neutrophil-to-lymphocyte ratio, ALT = Alanine transaminase, AST = Aspartate transaminase.

**Table 5:** Comparison of patients’ characteristics according to the existence of pneumonia.

Parameter/Pneumonia (N = 250)	Absent (N = 34)	Present (N = 216)	*p*
Sex (Female) (Nr., %)	22 (64.7%)	109 (50.5%)	0.141 ‡
Age (Median (IQR))	27 (23–38)	34 (24–43)	0.098 §
Workplace domain (Nr., %) (N = 239)			
Other (N = 213)	26 (81.3%)	187 (90.3%)	0.132 ‡
Healthcare (N = 26)	6 (18.8%)	20 (9.7%)	
Environment (Urban) (Nr., %) (N = 198)	17 (50%)	133 (61.6%)	0.258 ‡
Education (Nr., %)			
Primary (N = 22)	4 (13.3%)	18 (10.7%)	0.810 ‡
Secondary (N = 105)	17 (56.7%)	88 (52.4%)	
Academic (N = 39)	4 (13.3%)	35 (20.8%)	
No education (N = 32)	5 (16.7%)	27 (16.1%)	
History—Travel (Nr., %) (N = 249)	0 (0%)	2 (0.9%)	1.000 ‡
History—Antibiotic treatment (Nr., %)	12 (35.3%)	62 (28.7%)	0.426 ‡
Health insurance (Nr., %)	26 (76.5%)	156 (72.2%)	0.683 ‡
Symptoms			
Days—Onset—Admission (Median (IQR)) (N = 249)	5 (3.75–6.25)	5 (4–6)	0.794 §
Fever (Nr., %)	32 (94.1%)	210 (97.2%)	0.298 ‡
Days—Exanthema—Admission (Median (IQR)) (N = 243)	1 (1–2)	2 (1–3)	0.134 §
Complications (Nr., %)			
Respiratory failure	1 (2.9%)	40 (18.5%)	0.023 ‡
Encephalitis	0 (0%)	1 (0.5%)	1.000 ‡
Hospitalization period (Median (IQR))	4 (3–5)	4.5 (4–6)	0.054 §
Laboratory parameters (Median (IQR))			
Neutrophils (cells/μL)	3750 (2850–5475)	4500 (3400–5675)	0.232 §
Lymphocytes (cells/μL)	600 (400–700)	500 (400–800)	0.785 §
NLR	6.76 (5.45–10.56)	8.2 (5.47–11.68)	0.261 §
AST (UI/mL) (N = 241)	75 (57–230.5)	115.5 (60–218)	0.437 §
ALT (UI/mL) (N = 249)	98 (57–357.5)	158 (65–299.75)	0.354 §
ALT/AST (N = 241)	1.14 (0.89–1.47)	1.31 (0.96–1.69)	0.265 §
Antibiotic treatment (Nr., %)	16 (47.1%)	148 (68.5%)	0.019 ‡

‡ Fisher’s Exact Test, § Mann–Whitney U Test, N = Number of cases, IQR = Interquartile range, NLR = Neutrophil-to-lymphocyte ratio, ALT = Alanine transaminase, AST = Aspartate transaminase.

**Table 6:** Comparison of patients’ characteristics according to the existence of respiratory failure.

Parameter/Respiratory Failure (N = 250)	Absent (N = 209)	Present (N = 41)	*p*
Sex (Female) (Nr., %)	119 (56.9%)	12 (29.3%)	0.002 ‡
Age (Median (IQR))	32 (23–42)	37 (24.5–45)	0.049 §
Workplace domain (Nr., %) (N = 239)			
Other (N = 213)	175 (87.9%)	38 (95%)	0.269 ‡
Healthcare (N = 26)	24 (12.1%)	2 (5%)	
Environment (Urban) (Nr., %) (N = 198)	122 (58.4%)	28 (68.3%)	0.296 ‡
Education (Nr., %)			
Primary (N = 22)	17 (10.2%)	5 (16.1%)	0.560 ‡
Secondary (N = 105)	87 (52.1%)	18 (58.1%)	
Academic (N = 39)	35 (21%)	4 (12.9%)	
No education (N = 32)	28 (16.8%)	4 (12.9%)	
History—Travel (Nr., %) (N = 249)	2 (1%)	0 (0%)	1.000 ‡
History—Antibiotic treatment (Nr., %)	63 (30.1%)	11 (26.8%)	0.713 ‡
Health insurance (Nr., %)	154 (73.7%)	28 (68.3%)	0.565 ‡
Medical history—Measles (Nr., %)	1 (0.5%)	0 (0%)	1.000 ‡
Symptoms			
Days—Onset—Admission (Median (IQR)) (N = 249)	5 (3–6)	5 (4–7)	0.034 §
Fever (Nr., %)	204 (97.6%)	38 (92.7%)	0.127 ‡
Days—Exanthema—Admission (Median (IQR)) (N = 243)	1.5 (1–2)	2 (1–3)	0.390 §
Complications (Nr., %)			
Encephalitis	1 (0.5%)	0 (0%)	1.000 ‡
Hospitalization period (Median (IQR))	4 (3.5–6)	5 (4–6)	0.171 §
Laboratory parameters (Median (IQR))			
Neutrophils (cells/μL)	4100 (3100–5400)	5200 (4600–6800)	<0.001 §
Lymphocytes (cells/μL)	500 (400–700)	600 (300–850)	0.734 §
NLR	8 (5–11.16)	9 (7–17.08)	0.025 §
AST(UI/mL) (N = 241)	101 (52.5–225)	118 (76.5–164)	0.497 §
ALT (UI/mL) (N = 249)	134 (61.5–315)	157 (94.5–278)	0.737 §
ALT/AST (N = 241)	1.31 (0.96–1.66)	1.31 (0.88–1.74)	0.782 §
Antibiotic treatment (Nr., %)	129 (61.7%)	35 (85.4%)	0.004 ‡

‡ Fisher’s Exact Test, § Mann–Whitney U Test, N = Number of cases, IQR = Interquartile range, NLR = Neutrophil-to-lymphocyte ratio, ALT = Alanine transaminase, AST = Aspartate transaminase. Antibiotic treatment = antibiotics administered during hospitalization. History—Antibiotic treatment = antibiotics used in the 14 days before admission.

## Data Availability

The corresponding author can provide the data used in this study 641 upon reasonable request.

## Supplementary Materials

The following supporting information can be downloaded at: https://www.mdpi.com/article/10.3390/clinpract16010004/s1, Figure S1: ROC curve analysis for prediction of keratitis using NLR; Figure S2: ROC curve analysis for the prediction of keratitis using the obtained predictive score; Table S1: ROC curve analysis for prediction of keratitis using NLR; Table S2: Univariable and multivariabile binomial logistic regression models for the prediction of keratitis; Table S3: ROC curve analysis for the prediction of keratitis using the obtained predictive score; Table S4: Distribution of the patients with ocular lesions according to the type of ocular lesions and the predictive score for keratitis.

## Abbreviations

The following abbreviations are used in this manuscript:

ALT	Alanine transaminaze
AST	Aspartate transaminase
IQR	Interquartile range
NLR	Neutrophil-to-lymphocyte ratio
R_0_	Basic reproduction number
SD	Standard deviation
