# Supplementary material for: Patterns of Ocular Involvement and Associated Factors in Adult Measles: A Retrospective Study from a Romanian Tertiary Hospital
Source: Clin Pract. 2025 Dec 25;16(1):4. doi: 10.3390/clinpract16010004 (PMC12840547; doi:10.3390/clinpract16010004)
Supplement: Supplementary file 1 [file clinpract-16-00004-s001.zip › clinpract-4022041-supplementary.pdf]

Data analysis (supplementary)

ROC curve analyses were used to predict the existence of keratitis over other lesions in patients with ocular lesions. For each variable, performance of the prediction was measured using the AUC value with 95% confidence intervals, along with the significance value. The cut-off was chosen based on the highest Youden value observed, along with its sensitivity and specificity performance. A new binomial variable was created for NLR based on the found cut-off in the ROC curve.

Selection of the quantitative or binomial type of variable for NLR was based on a multivariable forward step-wise Wald binomial logistic regression model where the binomial variable had a higher level of prediction. Univariable and multivariable binomial logistic regression models were used to predict the existence of keratitis over other lesions in patients with ocular lesions. Models were tested for significance and goodness-of-fit. Performance of the prediction was measured as odds ratios with 95% confidence intervals along the significance value.

A prediction score was calculated based on odds ratios of significant parameters in the multivariable model. The score was tested and validated using ROC analysis and a contingency table (for PPV, NPV and accuracy).

Table S1. ROC curve analysis for prediction of keratitis using NLR

Parameter	AUC	95% C.I.	p	Cut-off	Se	Sp
**NLR**	0.661	0.544-0.779	**0.016**	9.75	54.7%	79.3%

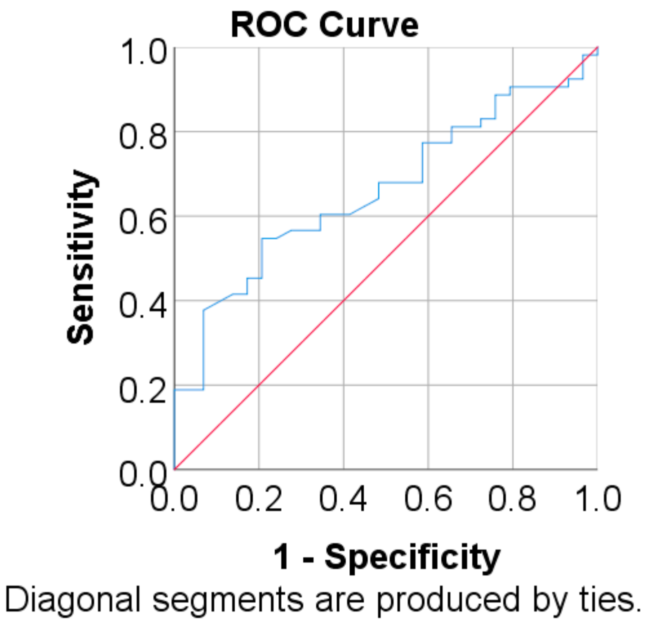

Figure S1. ROC curve analysis for prediction of keratitis using NLR

Data from **Table S1** and **Figure S1** show the ROC curve analysis for prediction of keratitis using NLR. The observed prediction is significant but with a poor power of prediction (AUC = 0.661, 95% C.I. = 0.544-0.779, **p=0.016)**; the obtained cut-off for NLR = 9.75 shows that patients with ocular lesions and a value for NLR higher or equal to 9.75 have a keratitis prediction sensitivity and specificity of 54.7% and 79.3%, respectively.

Table S2. Univariable and multivariabile binomial logistic regression models for the prediction of keratitis

Parameter	Univariable		Multivariable*	
	95% C.I.	p	95% C.I.	p
**NLR ≥ 9.75**	4.632 (1.623-13.219)	**0.004**	4.227 (1.364-13.1)	**0.012**
**Koplik sign**	6.923 (2.424-19.771)	**<0.001**	6.416 (2.126-19.365)	**0.001**

***Multivariable binomial logistic regression model, χ^2 (2) = 21.177, p<0.001, Nagelkerke R^2 = 0.313, Hosmer and Lemeshow test: p=0.508, overall accuracy = 73.2%**

Data from **Table S2** shows the univariable and multivariabile binomial logistic regression models for the prediction of keratitis. Selection of the quantitative or binomial type of variable for NLR was based on a multivariable forward step-wise Wald binomial logistic regression model where the binomial variable had a higher level of prediction. According to the results obtained in the multivariable model, both NLR and the existence of the Koplik sign are significant and independent predictors for the existence of keratitis; as such,

- Patients with an NLR value ≥ 9.75 have 4.227 times higher odds of having keratitis than other lesions (95% C.I. = 1.364-13.1) (**p=0.012)**;
- Patients with a positive Koplik sign have 6.416 times higher odds of having keratitis than other lesions (95% C.I. = 2.126-19.365) (**p=0.001)**.

In order to obtain a predictive score for keratitis, based on the predictors observed in **Table S2**, each odds ratio for the found variables is divided by the smallest observed odds ratio (here it is 4.227), thus attributing the number of points below for each observed factor (for each variable, the absence of the factor will be attributed with 0 points), while the score will be obtained by the sum of the points from the two factors:

- 1 point for an NLR value ≥ 9.75;
- 1.5 points for a positive Koplik sign.

Table S3. ROC curve analysis for the prediction of keratitis using the obtained predictive score

**Parameter**	**AUC**	**95% C.I.**	**p**	**Cut-off**	**Se**	**Sp**
**Score**	0.786	0.685-0.888	**<0.001**	2	49.1%	93.1%

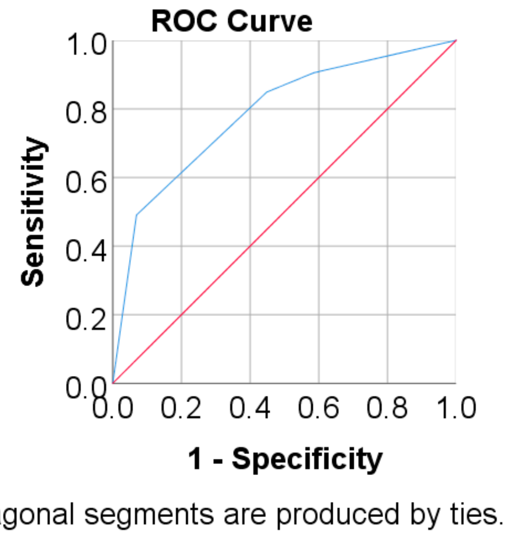

Figure S2. ROC curve analysis for the prediction of keratitis using the obtained predictive score

Data from **Table S3** and **Figure S2** show the ROC curve analysis for the prediction of keratitis using the obtained predictive score. The observed prediction is significant, with a good power of prediction (AUC = 0.786, 95% C.I. = 0.685-0.888, **p<0.001)**; the obtained cut-off score of 2 points (thus having both a positive Koplik sign and a value for NLR ≥ 9.75) shows these patients have a keratitis prediction sensitivity and specificity of 49.1% and 93.1%, respectively.

Table S4. Distribution of the patients with ocular lesions according to the type of ocular lesions and the predictive score for keratitis

Score/ Ocular lesions	Other lesions		Keratitis		p*
	Nr.	%	Nr.	%	
< 2 points	27	93.1%	27	50.9%	<0.001
≥ 2 points	2	6.9%	26	49.1%	

***Fisher's Exact Test**

In addition to the significant association (p<0.001), the contingency table in **Table S4** shows two important aspects. The positive predictive value of – 92.86% (95% C.I. = 76.85-98.07%) means that a patient with ocular lesions with a score of 2 points or higher (thus having both a positive Koplik sign and a value for NLR ≥ 9.75) has a 92.86% chance of having keratitis, and the negative predictive value of – 50% (95% C.I. = 42.99-57.01%) means that a patient with a score less than 2 points has a 50% chance of having other lesions. Overall accuracy: 64.63% (95% C.I. = 53.3-74.88%).
